# Integrated-boost IMRT or 3-D-CRT using FET-PET based auto-contoured target volume delineation for glioblastoma multiforme - a dosimetric comparison

**DOI:** 10.1186/1748-717X-4-57

**Published:** 2009-11-23

**Authors:** Marc D Piroth, Michael Pinkawa, Richard Holy, Gabriele Stoffels, Cengiz Demirel, Charbel Attieh, Hans J Kaiser, Karl J Langen, Michael J Eble

**Affiliations:** 1 Department of Radiation Oncology, RWTH Aachen University Hospital, Pauwelsstrasse 30, 52074 Aachen Germany; 2 Department of Nuclear Medicine, RWTH Aachen University Hospital, Pauwelsstrasse 30, 52074 Aachen Germany; 3 Institute of Neurosciences and Medicine, Research Centre Jülich, 52425 Jülich, Germany; 4 JARA (Jülich Aachen Research Alliance) Forschungszentrum Jülich GmbH Wilhelm-Johnen-Straße, 52428 Jülich, Germany

## Abstract

**Background:**

Biological brain tumor imaging using O-(2-[^18^F]fluoroethyl)-L-tyrosine (FET)-PET combined with inverse treatment planning for locally restricted dose escalation in patients with glioblastoma multiforme seems to be a promising approach.

The aim of this study was to compare inverse with forward treatment planning for an integrated boost dose application in patients suffering from a glioblastoma multiforme, while biological target volumes are based on FET-PET and MRI data sets.

**Methods:**

In 16 glioblastoma patients an intensity-modulated radiotherapy technique comprising an integrated boost (IB-IMRT) and a 3-dimensional conventional radiotherapy (3D-CRT) technique were generated for dosimetric comparison. FET-PET, MRI and treatment planning CT (P-CT) were co-registrated. The integrated boost volume (PTV1) was auto-contoured using a cut-off tumor-to-brain ratio (TBR) of ≥ 1.6 from FET-PET. PTV2 delineation was MRI-based. The total dose was prescribed to 72 and 60 Gy for PTV1 and PTV2, using daily fractions of 2.4 and 2 Gy.

**Results:**

After auto-contouring of PTV1 a marked target shape complexity had an impact on the dosimetric outcome. Patients with 3-4 PTV1 subvolumes vs. a single volume revealed a significant decrease in mean dose (67.7 vs. 70.6 Gy). From convex to complex shaped PTV1 mean doses decreased from 71.3 Gy to 67.7 Gy. The homogeneity and conformity for PTV1 and PTV2 was significantly improved with IB-IMRT. With the use of IB-IMRT the minimum dose within PTV1 (61.1 vs. 57.4 Gy) and PTV2 (51.4 vs. 40.9 Gy) increased significantly, and the mean EUD for PTV2 was improved (59.9 vs. 55.3 Gy, p < 0.01). The EUD for PTV1 was only slightly improved (68.3 vs. 67.3 Gy). The EUD for the brain was equal with both planning techniques.

**Conclusion:**

In the presented planning study the integrated boost concept based on inversely planned IB-IMRT is feasible. The FET-PET-based automatically contoured PTV1 can lead to very complex geometric configurations, limiting the achievable mean dose in the boost volume. With IB-IMRT a better homogeneity and conformity, compared to 3D-CRT, could be achieved.

## Introduction

In spite of intensive efforts to improve treatment strategies the prognosis of patients suffering from a Glioblastoma multiforme remains poor with a median survival time of 12-14 months [1,2]. Even though a radiation dose-response relationship could be demonstrated in clinical [3,4] as well as in experimental studies [5-8], no significant increase of survival could be achieved in randomized clinical trials. Although several Phase II-studies showed promising results [9,10], the RTOG 93-05 study failed to demonstrate a prognostic improvement for patients treated with a stereotactic boost in addition to the standard 60 Gy fractionated conformal radiotherapy with the alkylating agent carmustine (BCNU) [11]. The authors speculated that these results may be caused by the fact that glioblastomas (GBM's) are inherently infiltrating neoplasms. Another reason for the poor results of those studies, however, may be the inability of current imaging methods to adequately reflect the true extent of the tumors. Magnetic resonance imaging (MRI) is currently the method of choice for the diagnosis of primary brain tumors. The delineation between glioma and surrounding edema with MRI is unreliable since the tumor is not sharply demarcated and if in addition the blood-brain barrier remains intact. Therefore, it appears essential to base locally focused dose escalation concepts on more specific imaging methods, such as MR spectroscopy or Positron Emission Tomography (PET). Several data suggest that brain tumor imaging with PET using amino acids is more reliable than MRI to define the extent of cerebral gliomas [12-16]. O-(2-[^18^F]fluoroethyl)-L-tyrosine (FET) is a well established amino acid tracer for PET. Biological brain tumor imaging combined with inverse treatment planning for locally restricted dose escalation in patients with glioblastoma multiforme seems to be a promising approach.

The aim of this study was to compare inverse with forward treatment planning for an integrated boost dose application in patients suffering from a glioblastoma multiforme, while the auto-contoured biological target volumes are based on O-(2-[18F]Fluorethyl)-L-Tyrosin (FET)-PET and MRI data sets.

## Materials and methods

### Patients

Sixteen consecutive patients with a histologically proven supratentorial glioblastoma multiforme (WHO grade IV) were treated with an intensity-modulated radiotherapy comprising an integrated boost dose application (IB-IMRT). In addition a 3-dimensional conventional radiotherapy (3D-CRT) treatment plan was generated for dosimetric comparison. The selected patients were treated in our clinic from January 2008 to January 2009 within an ongoing prospective monocentric phase-II study. The mean age was 55.6 (36-73) years. Ten patients were male. The Karnofsky perfomance index was ≥ 70% in 15 patients. A gross total and partial resection could be achieved in 8 patients. The tumor was located in the right and left hemisphere in 4 and 12 patients. Half of the tumors were located in the frontal lobe, while the other half of patients showed an equally frequent location within the temporal or parietal lobe. The study was approved by the university ethics committee and federal authorities. All subjects gave written informed consent for their participation in the study.

### Target volume definition

After head fixation with a thermoplastic mask (Orfit^® ^Raycast^©^-HP mask system, mean target isocenter translation <2 mm [17]) a dedicated computer tomography (P-CT) with continuous slices of 2 mm thickness was made. An O-(2-F-18-Fluorethyl)-L-Tyrosin-PET (FET-PET) was performed in all 16 patients within 2 days after P-CT and within an interval of 11-20 days after surgical resection or biopsy of the tumor. Prior to FET-PET patients remained fasting for at least 6 h. PET images were acquired 15-40 min after intravenous injection of 200 MBq ^18^F-FET. The measurements were performed with an ECAT EXACT HR+ scanner (Siemens Medical Systems, Inc.) in 3-dimensional mode (32 rings; axial field of view, 15.5 cm) (details s [18]).

All patients received pre- and postoperative MRI's, performed in a 1,5 tesla MRI scanner with a standard head coil, which were integrated in the planning process. The MRI protocol consisted of a contrast enhanced T1-weighted, a T2-weighted and a FLAIR (fluid attenuation inversion recovery) sequence. All image data sets were reconstructed and imported into the Philips Pinnacle^3 ^irradiation treatment planning system (Version 8.0 m, Philips Medical Systems, Eindhoven, NL). The Philips Syntegra™ image registration tool was used to co-registrate the postoperative MRI and FET-PET to the native P-CT. From the three auto-registration methods available in Syntegra the Mutual Information (MI) method was used [19]. The image co-registration process was performed automatically. Finally the fusion results were assessed visually based on anatomic landmarks. The preoperative MRI was integrated side-by-side in the planning process.

Two clinical target volumes (CTV) were generated. For delineation of CTV1, defined as biological target volume from postoperative FET-PET imaging, an auto-contouring process was used.

The definition of the biological target volume with PET is a critical issue. Due to the limited spatial resolution of 5 mm it is not possible to define the exact tumor border on PET images. Tumor delineation based on the mean background activity such as the tumor/brain ratio appears to be an adequate approach for the problem of tumor definition in amino acid studies [20]. In a previous biopsy controlled study we found for tumor tissue a mean lesion-to-brain ratio of FET uptake of 2.6 ± 0.9 and 1.2 ± 0.4 for peritumoral tissue [15]. Others reported that best differentiation of tumor and non-tumoral tissue could be observed at tumor/brain ratios of 2.0 and 2.2 [16,21]. In the present study CTV1 was defined as the volume within a cut-off tumor-to-brain ratio (TBR) of ≥ 1.6. Since this threshold value is in the lower range the tumor volume is overestimated and is assumed to contain a safety margin of approx. 5 mm. Therefore no additional margin was given to between CTV1 and PTV1 (CTV1 = PTV1).

For generating the TBR a polygonal reference region was drawn over several axial P-CT slices, comprising a volume of 40-70 cm^3 ^from the contra-lateral cerebral hemisphere. Then the mean activity value of the normal brain reference area was multiplied by the cut-off value for automatic delineation of CTV1. Finally manual corrections were done, since the activity in blood vessels or postoperative extracerebral soft tissue could be above the cut-off value [22]. Venous structures, visible in the co-registrated MRI, were excluded. CTV-subvolumes comprising less than 3 voxels (FET-PET voxel size 2 × 2 × 2.4 mm) were deleted. We classified the shape of the target volume CTV1 into three categories: convex, concave and complex. The term "complex" describes a finger-shaped or cuttlefish like appearance. In addition the number of separate subvolumes for each CTV1 was considered for classification of target volume complexity.

The CTV2 was defined as the contrast-enhanced area from pre- and postoperative MRI including a safety margin of 2-3 cm. The margin was further extended to include the surrounding preoperative edema, individually adapted to organs at risk and osseous structures. The PTV2 was generated automatically by adding a 0.5 cm margin to the CTV2 and excluding CTV1.

A constant margin of 5 mm was added circumferentially around the PTV's to account for the penumbra of the radiation beams in 3D-CRT plans

### Dose prescription and treatment planning

For IMRT we used an integrated boost technique. The total dose was 72 Gy, prescribed to the ICRU Reference Point [23,24], resulting in daily fractions of 2.4 Gy for PTV1. A mean dose of 60 Gy was recommended for PTV2, resulting in daily fractions of 2 Gy.

For 3D-CRT we used a concomitant boost technique. The total dose of 72 Gy was prescribed to the ICRU Reference Point [23,24]. Dose calculations were separated in a dose prescription of 60 Gy for PTV1 and PTV2, and a dose prescription of 12 Gy for PTV1 alone, resulting in equal daily fractions of 2.4 Gy for PTV1 and 2 Gy for PTV2, compared to the integrated boost IMRT technique. Normal tissue dose constraints were 50 Gy (maximum point dose) for chiasm and optic nerves and 50 - 54 Gy for the brainstem. In table 1 the IMRT dose constraint values, setted initially for PTV's and OAR's, are shown.

**Table 1:** Dose constraint values, setted initially for PTV's and OAR's

Region of Interest	*Type*	Target Gy	% Volume
**PTV1**	max. dose	77.04	-

	uniform dose	72.00	-

	min DVH	68.40	95

**PTV2**	uniform dose	60.00	-

	max. dose	72.00	-

	max. DVH	67.50	5

	max. DVH	64.20	15

	max. DVH	63.00	25

**Brain**	max. DVH	25.00	40

	max. DVH	40.00	20

**Brainstem**	max. Dose	54.00	-

	max. DVH	50.00	30

**Chiasma**	max. dose	50.00	

**Optic nerves**	max. dose	50.00	

**lenses**	max. dose	5.00	

In all patients the OAR's were outside the PTV's. Despite a hypofractionated setting with single doses in PTV1 by 2.4 Gy the single doses in the OAR's were maximally 2 Gy, corresponding to a conventionally fractionation. So, from a radiobiologically point of view, the established constraints for the OAR's could be taken. The data for normal tissue complication probabilities are those described by Emami [25].

Plans were acceptable for both techniques when the given normal tissue constraints were fulfilled while the mean dose to PTV2 was 60 Gy.

For IMRT we used a step-and-shoot technique and 6-15 MeV photons for an Elekta Precise^© ^linear accelerator (multileaf collimator with leaves projecting to 1 cm at isocenter). The direct machine parameter optimization (DMPO, Pinnacle^© ^v8.0 m) algorithm was applied for inverse planning with a 2 cm^2 ^minimum segment area, five minimum segment monitor units and a maximum number of 100 segments. The dose grid size includes the PTV's, organs at risk and scalp and additionally 1-4 cm tissue in all directions. The beam arrangements were determined by the size and location of the tumor and the corresponding PTV's. No restrictions were given for the number of beams or angles or whether noncoplanar beams could be used. For 3D-CRT we used 2-6 beams to cover PTV1 and also PTV2 (table 2).

**Table 2:** Summerized plan information

	mean (range)	
	**IMRT**	**3D-CRT**

Monitor Units (MU)	606 (483-845)	482 (256-804)

Beam Number	7 (5-9)	9 (4-12)*

Segments	91 (70-100)	-

Wedge Number	-	3 (0-5)

Beam energy (MeV)	6-15	6-15

(*summarized beam number for covering PTV1 and PTV2)

### Plan comparison

Treatment plan intercomparisons were performed using the following criteria: mean, minimum and maximum doses, Inhomogeneity Index (II), Conformity Index (CI) and Equivalent Uniform Dose (EUD)

### Inhomogeneity Index (II) and Conformity Index (CI)

Two indices served to characterize homogeneity and conformity:

◦ Inhomogeneity Index [26]   II = (D_max _- D_min_)/D_mean_

D_max_: maximum PTV dose; D_min_: minimum PTV dose; D_mean_: mean PTV dose;

◦ Conformity Index [27]   CI = PTV_PIV_^2^/PTV * PIV

PTV_PIV_: PTV volume covered by 95% of the prescription dose; PIV: total volume covered by 95% of the prescription dose.

### EUD (Equivalent Uniform Dose)

The EUD, defined as the biologically equivalent dose that, if given uniformly, will lead to the same effect in the tumor volume or the normal tissues as the actual nonuniform dose distribution, could be, based on Niemierko [28,29], defined as:

N: number of voxels in the anatomic structure of interest; d_i_: dose in the i'th voxel; a: tumor or tissue-specific parameter that describes the dose volume effect.

In Pinnacle^3 ^IMRT, which is used for EUD-calculation, the equation is slightly modified to allow voxels to be only partially included in a region of interest [30] as:

v: fraction of the region of interest that is occupied by voxel "i".

In this analysis the tumor or tissue-specific parameter "a", that describes the dose volume effect, was taken, based on Burman, as follows: a = -10 for malignant glioma, a = 4 for brain, a = 6.25 for brain stem, a = 4 for chiasm and optic nerves [31-33].

### Statistics

Statistical analysis was performed using the SPSS 17.0 (SPSS^®^, Chicago, Ill) software. The Wilcoxon's matched-pair's test was applied to determine statistical differences between the dose-volume-load calculated with the IB-IMRT- versus 3D-CRT-plans and also to determine statistical differences between mean doses and EUD's in the IMRT- and 3D-CRT-plans. Values are expressed as mean ± standard deviation or as mean value and the range of the values. All p-values reported are two-sided and p < 0.05 is considered significant.

## Results

### Target subvolume number and shape

After the described auto-contouring process, based on FET-PET data for PTV1 most patients revealed a complex shape together with multiple subvolumes. Looking on the number of these subvolumes only in 4 patients a sole subvolume was defined, while in 5 and 6 patients 2 and 3 subvolumes appeared, respectively. In one patient a total of 4 subvolumes resulted from the auto-contouring process. In respectively 2, 8 and 6 patients the automatically generated PTV1 had a convex, concave and complex shape (table 3).

**Table 3:** Target volume characteristics

Target volumes	PTV1 (= CTV1 *)	12.1 ± 18.6 ccm
	**PTV2**	**175.2 ± 54.4 ccm**

		

PTV1 subvolume number		patient number
	1	4
	2	5
	3	6
	4	1

PTV1 geometry single form and/or subvolume configuration	convex	2
	concave	8
	complex	6

Tumor/Brain ratio (in PTV1)	Mean	2.1 (1.7-2.9)
	max	3.3 (2.0-4.9)

(* CTV1 is equal to PTV1, s. *Target volume definition*)

Based on the described dose prescription of 72 Gy as point dose to PTV1, patients with a single subvolume (n = 4) had a mean dose of 70.6 (69.2-71.5) Gy to PTV1. In patients with 3 (n = 6) or 4 (n = 1) subvolumes the mean dose decreased to 67.6 (66.0-68.5) Gy. According to the complexity in the shape of the target volumes an equal decrease of mean dose for PTV1 was observed. For convex shaped PTV1 a mean dose of 71.28 (66.11-73.07) Gy resulted, while in patients with a complex shape the mean dose decreased to 67.70 (59.72-72.99) Gy (table 4, 5).

**Table 4:** Mean, min. and max. dose (IMRT) in correlation to the subvolume-number in PTV1

			IMRT		
	**number of subvolumes**	**n**		**dose**	**SD**

**PTV 1**	overall	16	**mean**	**68.76**	± 1.88
			min	61.07	± 3.31
			max	73.14	± 0.98

	1	4	**mean**	**70.60**	± 1.01
			min	63.61	± 3.84
			max	73.56	± 0.93

	2	5	**mean**	**68.50**	± 1.91
			min	60.54	± 3.47
			max	73.72	± 1.13

	3/4	7	**mean**	67.56	± 0.94
			min	59.99	± 2.46
			max	71.94	± 1.32

**Table 5:** Mean, min. and max. (IMRT) in correlation to the PTV1-configuration

			IMRT		
	**PTV1-configuration**	**n**		**dose**	**(SD)**

**PTV 1**	overall	16	**mean**	**68.76**	± 1.88
			min	61.07	± 3.31
			max	73.14	± 0.98

	Convex	2	**mean**	**71.28**	± 0.35
			min	66.11	± 3.56
			max	73.05	± 0.72

	Concave	8	**mean**	**68.66**	± 1.79
			min	60.82	± 3.31
			max	72.81	± 1.85

	complex	6	**mean**	**67.70**	± 0.98
			min	59.72	± 1.58
			max	72.99	± 1.03

### Inhomogeneity and conformity

Using the inverse planning technique for IB-IMRT the dose inhomogeneity within PTV1 (HI: 0.17 vs. 0.24, p = 0.02) and within PTV2 (HI: 0.34 vs. 0.54, p < 0.01) decreased significantly, compared to 3D-CRT.

The dose conformity for PTV1 (CI: 0.35 vs. 0.14, p < 0.01)and for PTV2 (CI: 0.64 vs. 0.5, p < 0.01) was significantly improved with IB-IMRT (table 6).

**Table 6:** Inhomogeneity Index and Conformity Index for PTV1 and 2 in IMRT versus 3D-CRT

	Inhomogeneity Index					Conformity Index				
	**IMRT**		**3D-CRT**		**p**	**IMRT**		**3D-CRT**		**p**

	**Mean**	**SD**	**mean**	**SD**		**mean**	**SD**	**mean**	**SD**	

**PTV1**	0.17	± 0.05	0.24	± 0.12	**0.02**	0.35	± 0.12	0.14	± 0.1	**<0.01**

**PTV2**	0.34	± 0.54	0.54	± 0.13	**<0.01**	0.64	± 0.07	0.50	± 0.13	**<0.01**

### Mean dose, minimum and maximum dose

The averaged mean dose for PTV2 was slightly, but significantly lower (60.68 ± 0.63 Gy vs. 61.00 ± 0.78 Gy, p = 0.03) after inverse treatment planning with IB-IMRT. For PTV1 the mean dose did not differ significantly (68.76 ± 1.88 Gy vs. 64.40 ± 2.79 Gy, p = 0.61). The minimum dose within PTV1 (61.1 Gy vs. 57.4 Gy, p = 0.02) and within PTV2 (51.4 Gy vs. 40.9 Gy, p < 0.01) increased highly significant after inverse treatment planning. Looking on the dose-volume-load to critical organs only the mean dose to the brain increased significantly (25.6 Gy vs. 22.9 Gy, p < 0.01) (table 7).

**Table 7:** Mean, min., and max. doses for PTV's and OAR's in IMRT versus 3D-CRT

		IMRT		3D-CRT		p
		**Dose**	**SD**	**dose**	**SD**	

**PTV 1**	mean	68.76	± 1.88	64.40	± 2.79	0.61
	min	61.07	± 3.31	57.39	± 6.79	**0.02**
	max	73.14	± 0.98	73.94	± 1.88	0.1

**PTV 2**	mean	60.68	± 0.63	61.00	± 0.78	**0.03**
	min	51.40	± 3.44	40.89	± 7.03	**<0.01**
	max	71.90	± 1.51	73.68	± 2.64	**0.01**

**Brain**	Mean	25.57	± 3.24	22.90	± 4.31	**<0.01**

**Brainstem**	mean	13.76	± 8.74	13.37	± 9.25	0.79
	max	37.04	± 20.2	36.56	± 20.15	0.77

**Chiasm**	mean	18.51	± 12.56	15.83	± 13.33	0.14
	max	28.16	± 17.9	23.56	± 16.84	0.07

**Optic nerve rt**.	mean	8.48	± 6.49	7.57	± 8.66	0.64
	max	15.2	± 12.04	12.19	± 12.43	0.14

**Optic nerve lt**.	mean	13.02	± 11.94	13.50	± 14.33	0.79
	max	18.99	± 16.39	18.20	± 17.69	0.69

### EUD

The averaged mean EUD for PTV2 was significantly improved (59.92 ± 0.95 Gy vs. 55.3 ± 4.33 Gy, p < 0.01) if planned with IB-IMRT vs. 3D-CRT. In addition the EUD for PTV1 was slightly improved (68.3 ± 1.93 Gy vs. 67.3 ± 2.85 Gy, p = 0.2) after IB-IMRT. The EUD for the brain was equal with both two planning techniques (41.7 ± 3.12 Gy vs. 41.6 ± 2.16 Gy) (s. table 8).

**Table 8:** Mean dose and EUD for PTV's and OAR's in IMRT versus 3D-CRT

	Mean Dose					EUD				
	**IMRT**		**3D-CRT**		**p**	**IMRT**		**3D-CRT**		**p**
	**mean**	**SD**	**mean**	**SD**		**mean**	**SD**	**mean**	**SD**	

**PTV 1**	68.76	± 1.88	64.40	± 2.79	0.61	68.34	± 1.93	67.29	± 2.85	0.2

**PTV 2**	60.68	± 0.63	61.00	± 0.78	**0.03**	59.92	± 0.95	55.30	± 4.33	**<0.01**

**Brain**	25.57	± 3.24	22.90	± 4.31	**<0.01**	41.57	± 2.16	41.73	± 3.12	0.69

**Brainstem**	13.76	± 8.74	13.37	± 9.25	0.79	21.83	± 11.91	22.49	± 12.94	0.48

**Chiasm**	18.51	± 12.56	15.83	± 13.33	0.14	19.64	± 13.2	16.95	± 13.71	0.12

**Optic nerve rt**.	8.48	± 6.49	7.57	± 8.66	0.64	10.2	± 7.76	8.81	± 9.49	0.41

**Optic nerve lt**.	13.02	± 16.94	13.50	± 14.33	0.79	14.07	± 12.74	14.57	± 14.65	0.78

## Discussion

In malignant gliomas distant tumor spread is rare and more than 80% of recurrences were found within a rim of 2-3 cm around the initial tumor site [34,35]. Therefore, it seems promising to escalate the radiation dose. In the past, several authors reported improved survival data from non-randomized, retrospective dose escalation trials [10,36,37]. These data should be interpreted cautiously because of a potential bias from patient selection [38-40]. In a RTOG multicenter phase-I-trial (RTOG 98-03) dose escalation was conducted using 3D-conformal irradiation [41]. In this trial a four step dose escalation strategy from 66 to 84 Gy was applied and median survival increased from 11.6 to 19.3 months in patients with a boost target volume smaller than 75 ccm. However, with boost target volumes ≥ 75 ccm the improvement was markedly smaller (8.2 vs. 13.9 months). No benefit was seen in progression free survival.

None of the randomized trials could demonstrate an improvement in median survival after locally restricted dose escalation. Souhami used a stereotactic boost technique [11], Laperriere [42] and Selker [43] used brachytherapy in addition to external beam radiotherapy with 60 Gy. Souhami addressed, that the results from the RTOG 93-05 trial were not completely surprising, because glioblastomas are inherently infiltrating neoplasms. Considering that delineation of tumor volumes in treatment planning was based on morphological imaging, they discussed, that "biopsy and magnetic resonance spectroscopy analyses have demonstrated significant microscopic tumor extension beyond the contrast-enhancing lesion, thereby limiting the effectiveness of focal radiotherapy".

MRI is highly sensitive in detecting brain tissue abnormalities. In a biopsy-controlled trial Pauleit obtained a 96% sensitivity to detect glioma tissue [15]. But the specificity was only 53%. Better tumor brain delineation became possible with the use of PET (Positron-Emission-Tomography) imaging with radio-labeled amino acids, like O-(2-F-18-Fluorethyl)-L-Tyrosin (FET) [18]. The use of FET-PET in addition to MRI yields a sensitivity of 93%, similar to MRI alone, but a markedly improved specificity of 94% [15].

Therefore, it is straightforward to integrate FET-PET imaging in dose escalation irradiation strategies. Several authors could already demonstrate the feasibility of this approach and mentioned the estimated positive impact [44-46]. The process of automatic delineation of the PET-positive area as biological target volume, done in our planning study, prevents the known problem of interobserver variations [47] but leads to a pronounced irregularity in target volume shape. After auto-contouring the PET-positive areas in our study with a cut-off value of 1.6, 37.5% of the patients revealed a very complex target shape, comprising multiple separate sub volumes, half of them cuttlefish-like shaped and mostly arranged around the surgical cave.

Irradiation with intensity-modulated dose application led to an improvement in target coverage compared with 3D-CRT in different tumor entities, i.e. head and neck [48], lung [49], breast [50], prostate [32,51,52] or other [53]. For radiotherapy of glioblastomas the feasibility and efficacy of IMRT planning with a simultaneous boost could be shown by Chan et al. [54]. Narayana et al. found no improvement in target coverage using IMRT in high-grade gliomas in comparison with 3D-CRT. Nevertheless, the normal brain, which received a dose of ≥ 18 and ≥ 24 Gy as well as the mean dose to the brainstem could be reduced with IMRT [55]. In contrast, MacDonald demonstrated in a similar analysis an improved target coverage and also confirmed reduced radiation dose to the brain, brainstem and optic chiasm [56]. Also Hermanto showed an improved target conformity using an IMRT vs. 3D-CRT planning for high-grade gliomas [57]. A locally restricted integrated dose escalation was not considered in these analyses.

**Figure 1:**
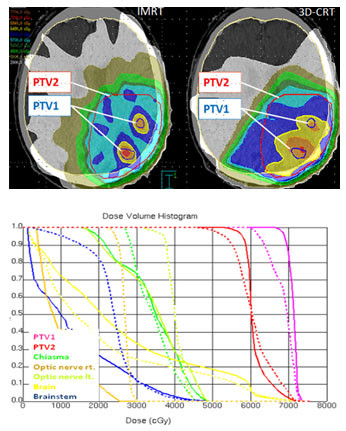
**a) Isodose distribution (dose wash) for IMRT and 3D-CRT-planning**. **b) Dose-volume-histograms for IMRT and 3D-CRT in comparison (IMRT: aligned, 3D- CRT: dashed).**

In our setting, using an integrated complex boost volume a significantly better conformity could be achieved with IMRT for both planning target volumes, PTV1 (0.35 vs. 0.14; p < 0.01) and PTV2 (0.64 vs. 0.5, p < 0.01). The dose inhomogeneities for PTV1 and PTV2 decreased significantly with IMRT (table 6, figure 1a, b).

In addition to the prescribed dose within PTV1 a dose of 60 Gy as mean dose for PTV2 was required as equally rated first level priority. In contrast to the ICRU 50/62 reports [23,24], which limits the recommended dose range between 95 and 107% of the prescribed dose to PTV2, a dose of 120% was accepted as essential default value to achieve a point dose prescription of 72 Gy within PTV1. To limit at least the integral dose to PTV2 a mean dose of 60 Gy with a minimum dose of 95% - as second level priority - was required for plan acceptance. The resulting mean doses for PTV2 were acceptable for IB-IMRT planning (60.68 Gy ± 0.63) and 3D-CRT planning (61.00 Gy ± 0.78). Using a prescription dose of 72 Gy as point dose to PTV1, the mean dose to PTV1 was less than the prescribed dose in all patients. After 3D-CRT planning a mean dose of 64.4 ± 2.79 Gy could be obtained and after IMRT planning the mean dose averaged over all patients was 68.76 ± 1.88 Gy to PTV1 (table 7).

**Figure 2:**
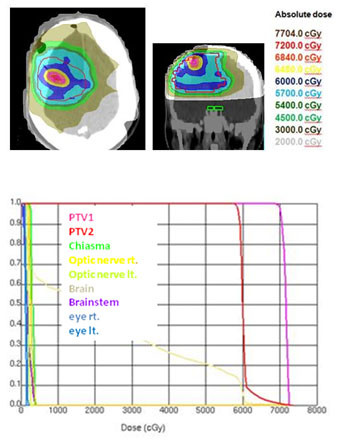
**a) Dose wash for IMRT**. Explanation of a convex configuration of PTV1 (one FET- subvolume) with a mean dose of 70.5 Gy. b) Dose-volume-histogram (IMRT) for a convex configuration of PTV1 (one FET-subvolume) with a mean dose of 70.5 Gy.

**Figure 3:**
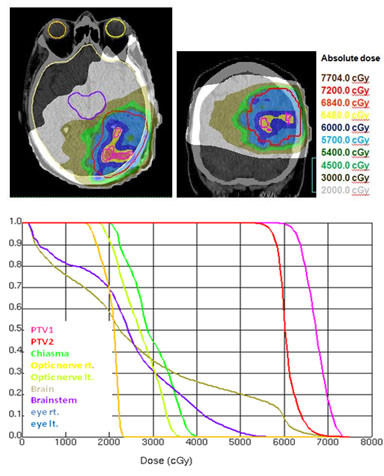
**a) Isodose distribution (dose wash) for IMRT**. Explanation of a concave configurationof PTV1 (3 FET-subvolumes) with a mean dose of 68.0 Gy.** b)Dose-volume-histogram (IMRT) for a concave configuration of PTV1 (3 FET-subvolumes) with a mean dose of 68.0 Gy.**

**Figure 4:**
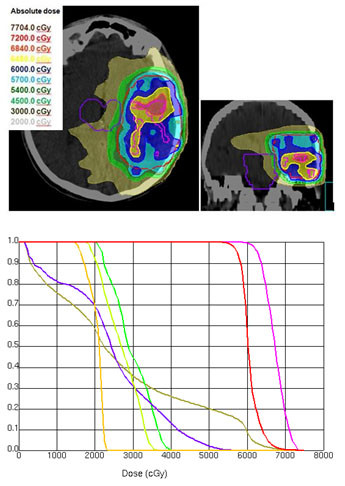
**a) Isodose distribution (dose wash) for IMRT**. Explanation of a complex. configuration of PTV1 (with 2 FET-subvolumes) with a mean dose of 67.2 Gy. **b) Dose-volume-histogram (IMRT) for a complex configuration of PTV1 (with 2 FET-subvolumes) with a mean dose of 67.2 Gy.**

Using an integrated boost technique for patients with high-grade gliomas, Thilmann could deliver an escalated mean dose of 75 Gy to the enhancing lesion in MRI (PTV1) and a mean dose of 60 Gy to the surrounding clinical risk area (PTV2) [58]. The authors allowed a dose delivery of more than 107% of the prescribed dose to 13.9% of the PTV2 volume. The maximum dose constraints for chiasm and optic nerves (52 Gy) were slightly increased compared to our study. A marked difference to our study was the MRI based delineation of PTV1, resulting in convex shaped singular target volumes. The shape and number of subvolumes of auto-contoured target volumes in our study was markedly more complex and had an impact on the mean dose value for PTV1 (tables 4 and 5, figures 2a, b, 3a, b, 4a, b). In patients with a singular PTV1 (n = 4) a mean dose of 70.6 Gy was achievable. But in patients with 3 and 4 subvolumes of PTV1 the mean dose decreased to 67.6 Gy.

The averaged minimum dose to PTV2 after 3D-CRT planning was 40.9 Gy (68% of the required dose) and thus not acceptable for most of the patients. After IB-IMRT planning the mean minimum dose to PTV2 increased to 51.4 Gy (86% of the required dose). The minimum dose was located 1.5-2 cm distant to the contrast-enhanced area in MRI and thus acceptable for treatment [41,59]. According to Tome and Fowler a minimum dose or "cold dose" lower than the prescribed dose by substantially more than 10% can be detrimental in tumor control [60]. In addition, Niemierko emphasized, that a "cold spot" cannot be compensated by any dose delivered to the rest of the target volume [28]. Unlike the mean dose, the equivalent uniform dose (EUD) concept includes the impact of dose inhomogeneities and volumetric effect [61]. The EUD is the homogeneous dose inside an organ that has the same clinical effect as a given, arbitrary dose distribution [62]. The EUD concept allows reducing a complex three-dimensional dose distribution into a single metric value [33,62]. Niemierko pointed out, that for relatively small dose inhomogeneities the mean dose might be a good approximation to EUD [28]. Furthermore, the authors explained that the minimum target dose can significantly underestimate the dose actually delivered, if the cold spot is very small. Considering the EUD concept, marked differences were evident in our study for PTV2. The EUD for PTV2 after 3D-CRT was significantly lower (55.3 Gy and 59.92 Gy, p < 0.01), while for PTV1 no significant difference was obtained.

For the OAR's only the EUD values for the brainstem differed significantly (25.6 Gy IB-IMRT; 22.9 Gy 3D-CRT, p < 0.01) (table 8). In contrast to the increase in mean dose, the EUD values for the brain were not significantly different (41.6 Gy for IB-IMRT and 41.7 Gy for 3D-CRT (p = 0.7)).

## Conclusion

Auto-contouring of the integrated boost volume resulted in complex target volume shapes. With the given constraints, the dose prescription to PTV1 (72 Gy), combined with a limited mean dose to PTV2 (60 Gy) could be achieved. Nevertheless a mean dose of 72 Gy to PTV1 could not be realized, neither with 3D-CRT nor with the IB-IMRT. Comparing both techniques, IB-IMRT provided several improvements. IB-IMRT led to a significantly better homogeneity and conformity, compared to 3D-CRT. The mean dose and the EUD values for PTV2 were inacceptable low with 3D-CRT, which would decrease tumor control. The EUD concept seems to be very useful for inverse planning, because the complex dose distribution can be reduced to one single parameter and also volume parameters and biological effects are taken into account. Further plan comparisons are simplified. The prognostic impact of this technique based on MR- and FET-PET imaging for patients with glioblastomas will be evaluated in an ongoing prospective phase-II trial in our clinic.

## Competing interests

The authors declare that they have no competing interests.

## Authors' contributions

MDP has made substantial contributions to the conception, acquisition of data, analysis and interpretation of data and drafted the manuscript. MP has been involved in acquisition of data and revised the manuscript. RH has been involved in acquisition of data and revised the manuscript. GS has been involved in acquisition of data and revised the manuscript. CD has been involved in acquisition of data and revised the manuscript. CA has been involved in acquisition of data and revised the manuscript. HJK has been involved in acquisition of data and revised the manuscript. KJL has made substantial contributions to the conception, acquisition of data, analysis and interpretation of data and revised the manuscript. MJE has made substantial contributions to the conception, acquisition of data, analysis and interpretation of data and revised the manuscript.
